# Characterization of the Newly Established Homoharringtonine- (HHT-) Resistant Cell Lines and Mechanisms of Resistance

**DOI:** 10.1155/2022/2813938

**Published:** 2022-08-30

**Authors:** Fenglin Li, Qing Ling, Chao Hu, Huafeng Wang, Wenle Ye, Xia Li, Xiang Zhang, Xiangjie Lin, Wenwen Wei, Xin Huang, Yu Qian, Haihui Zhuang, Jie Jin, Ying Lu

**Affiliations:** ^1^Department of Hematology, The Affiliated People's Hospital of Ningbo University, Ningbo, China; ^2^Department of Hematology, The First Affiliated Hospital, College of Medicine, Zhejiang University, Hangzhou, Zhejiang, China; ^3^Zhejiang Provincial Key Lab of Hematopoietic Malignancy, Zhejiang University, Hangzhou, Zhejiang, China

## Abstract

Homoharringtonine- (HHT-) based HHT, aclarubicin, and cytarabine (HAA) induction regimen is the first-line therapy for nonelder acute myeloid leukemia (AML) patients in China. However, drug resistance is a new challenge, and little attention has been devoted to excavating resistant mechanisms. This study used the classic method to construct six HHT-resistant cell lines with a gradually increasing resistance index (RI) to discover HHT drug resistance mechanisms dynamically. After HHT resistance, the cell growth rate decreased, cell cycle delayed, and P-glycoprotein (p-gp, CD243) expression levels increased. Furthermore, we explored the changes in transcriptomics between HHT-sensitive and HHT-resistant cells using RNA-sequence. Through Kyoto Encyclopedia of Genes and Genomes (KEGG), Gene Ontology (GO), and hub gene analyses, we found that immune activity, especially G-protein coupled receptor (GPR) and related molecules, may mediate HHT resistance. Moreover, Calcitonin Receptor-Like (CALCRL) and *G* Protein Subunit Alpha I1 (GNAI1), which belong to GPRs, were stimulated in HHT-resistant cell strains in vitro and vivo, indicating that they may play a critical role in HHT resistance. In addition, these two genes have prognostic significance for AML patients. Taken together, we successfully constructed HHT-resistant cell lines with dynamic RIs and explored the resistance mechanisms, which will help identify new drugs for HHT-resistant AML patients.

## 1. Introduction

Acute myeloid leukemia (AML) is the most common form of acute leukemia in adults and is characterized by stem cell transcriptional dysregulation and leads to a block in the differentiation [1]. Nowadays, advances in molecular biology and genetics have spurred advancements in diagnosis and prognosis in AML (2). However, the traditional treatment for AML, the DA or IA program, has not changed significantly over the past 40 years and is accompanied by a poor prognosis. Although many researchers have attempted to optimize traditional chemotherapy, the overall survival is still not ideal [2–4]. The main reasons for poor prognosis in AML patients are primary drug resistance and disease relapse. Moreover, the presence of drug-resistant leukemia cells is the key cause of relapse [5]. Therefore, characterizing drug-resistant leukemia cells and exploring resistance mechanisms are effective ways to improve AML prognosis.

Homoharringtonine (HHT) is a cytotoxic alkaloid that was initially extracted from *Cephalotaxus hainanensis*, and it has significant antileukemic effects in AML, chronic myeloid leukemia (CML), and myelodysplastic syndrome (MDS) [6]. One phase 3 clinical study showed that the complete remission (CR) (73% versus 61%, *p*=0.00108) and the 3-year event-free survival (EFS) (35.4% versus 23.1%, *p*=0.00023) in the HAA (HHT plus aclarubicin and cytarabine) treatment group were better than those in the DA (daunorubicin plus cytarabine) group [7]. The results suggest that the HAA regimen could be an alternative induction treatment for untreated AML patients, particularly those with favorable and intermediate cytogenetics. According to these results, HHT-based “HAA” program is the first-line therapy for nonelder AML patients in China [8]. The effectiveness of HAD (HHT + cytarabine + daunorubicin) program was also confirmed by another team in China [9]. However, the RFS of patients with HAA regimen was similar to that of DA regimen, and drug resistance has also become a challenge with the widespread use of HHT in AML. Exploring the resistance mechanism and related targets is crucial for refractory and relapsed AML patients who rely on HHT-based treatment.

Studies on the mechanisms of cytotoxicity and resistance to chemotherapy have been based on the development and analysis of resistant cancer cell lines [10]. For hematological malignancies, drug-resistant cell lines have been successfully constructed by increasing the concentration of a specific drug. The first successfully constructed drug-resistant cell line in hematology was K562 in 1983, which was exposed to increasing vincristine doses from 3 nM to 60 nM (12). Subsequently, drug-resistant cell lines for mantle cell lymphoma [11], multiple myeloma [12], and AML [13–15] have been successfully constructed.

Based on the above-mentioned background, we aimed to establish HHT-resistance AML cell lines with different RIs via exposure to gradually increasing HHT concentrations. After 15 months cultivated with HHT, 6 HHT-resistant cell lines were stabilization-established. Then cell characteristics including cell growth, apoptosis, cell cycle, and antigen p-gp were investigated to explore the biological behaviors difference between resistant cells and sensitive cells. From RNA-sequence data, we found that immune activity, especially G-protein coupled receptor and related molecules, such as CALCRL and GNAI1, may mediate HHT resistance. However, we have not further explored how CALCRL and GNAI1 mediate HHT resistance and the mechanism of resistance, and further exploration is needed in the future. The role of HHT in AML drug resistance is also explored from metabolomics or proteomics in the future.

## 2. Methods

### 2.1. Cell Culture

AML cell lines THP-1 (CVCL_0006), HL-60 (CVCL_A794), NB4 (CVCL-0005), Kasumi-1 (CVCL_0589), MOLM-13 (CVCL_2119), MV4-11 (CVCL_0064), KG-1 (CVCL_0374), and U937 (CVCL_0007) were purchased from American Type Culture Collection (ATCC), and OCI-AML3 (CVCL_1844) was purchased from Deutsche Sammlung von Mikroorganismen und Zellkulturen (DSMZ). THP-1, HL-60, OCI-AML3, NB4, Kasumi-1, KG-1*α*, and U937 were cultured in RPMI-1640 medium (HyClone, UT, USA); MOLM-13 and MV4-11 were cultured in IMDM medium (Corning Cellgro, USA), and supplemented with 10% fetal bovine (Thermo Fisher Scientific, Gibco, USA) at 37°C with 5% CO_2_. All cells were passaged less than 20 times and had cell identification certificates.

### 2.2. Cell Proliferation Assay

Proliferation and viability of AML cells were measured by CellTiter 96® AQueous One Solution Cell Proliferation Assay (Promega, USA). Briefly, THP-1, HL-60, OCI-AML3, NB4, Kasumi-1, KG-1, U937, MOLM-13, MV4-11, and resistant strains were inoculated into 24 wells at a final concentration of 1 × 10^5^ ml and treated with the indicated concentrations of HHT for 48 hours. Then 100 *μ*L cell supernatant was transferred into 96-well plates and 10 *μ*L CellTiter 96® Aqueous One Solution Cell Proliferation Assay was added per well. Absorbance was measured by a microplate reader (Thermo Scientific Varioskan® Flash, USA) at 490 nm to determine the OD value after 4 hours. The GraphPad software (version 6) calculated the cell proliferation rate and half-maximal inhibitory concentration (IC_50_) value according to the OD.

### 2.3. Construction of HHT-Resistant Cells with Dynamic Resistance Index (RI)

MV4-11 and MOLM-13 cells were treated with gradually increasing HHT concentrations (1 nmol/L to 50 nmol/L). The concentration of HHT was increased at approximately 2-week to 4-week intervals and maintained until cells grew normally in a complete growth medium in the presence of different concentrations of HHT for two weeks. Cells that grew normally in medium with 10% fetal bovine containing 10 nmol/L, 30 nmol/L, and 50 nmol/L HHT were preserved. These cells were designated as MV4-11 R10, MV4-11 R30, MV4-11 R50, MOLM-13 R10, MOLM-13 R30, and MOLM-13 R50. Then, the IC_50_ value of those cells was calculated and the RI of those cells was calculated with the following formula: IC_50_ (resistance)/IC_50_ (sensitive).

### 2.4. Flow Cytometry Analysis

To examine the cell apoptosis, MV4-11 and MOLM-13 sensitive and resistant cells were inoculated into 24 wells at a final density of 1 × 10^5^/ml. Then cells were collected and washed twice with 1× phosphate-buffered saline (1× PBS) and inoculated into 300 *μ*L 1× binding buffer with 10 *μ*L Propidium (PI) and 5 *μ*L Annexin-V for 30 minutes (Cat: AP101, MULTISCIENCES, Hangzhou, China). For cell cycle analysis, cells were seeded in 24 wells at a final concentration of 3 × 10^5^/ml cells per well and collected and fixed with precooled 75% ethanol at 4°C overnight. The next day, cells were washed twice with 1× PBS. Flow cytometry was utilized to detect the DNA content after PI (Cat: CCS012, MULTISCIENCES, Hangzhou, China) staining for about 30 minutes. For p-gp expression analysis, cells were inoculated into 300 *μ*L of 1× PBS with 10 *μ*L of p-gp antibody (RRID: AB_396548, BD, USA) labeled with APC for 30 minutes. Flow cytometry was performed and analyzed on ACEA NovoCyte (ACEA, USA).

### 2.5. Mouse Xenograft Model

Male BALB/c nude mice (5 weeks old) were obtained from Shanghai Experimental Animal Center of the Chinese Academy of Sciences (Shanghai, China). After one week of acclimatization, mice were subcutaneously injected with 1 × 10^6^ MV4-11 and MV4-11 R50 cells (suspended in 100 *μ*l 1× PBS), respectively. Tumor volumes were evaluated using the following formula: 1/2 × length × width^2^. After 7∼11 days, tumor volume reached 100–200 mm^3^, mice were randomly divided into MV4-11 group (*n* = 4) and MV4-11R group (*n* = 5), and HHT was administered by intraperitoneal injection every day at 0.5 mg/kg body weight for 7 days. Tumor volumes were measured twice daily using the caliper. All animal experiments were reviewed and approved by the Ethics Committee for Laboratory Animals of the First Affiliated Hospital, College of Medicine, Zhejiang University (Hangzhou, China) (reference number: 20201522).

### 2.6. Clinical Patients

178 AML patients between March 2010 and June 2014 with detailed diagnosis and treatment information were included in this study. Patients with acute promyelocytic leukemia (APL) and patients undergoing bone marrow transplantation were excluded. The clinical characteristics of the patients are summarized in Table 1. WHO classification, conventional cytogenetic banding assay, and molecular analyses were performed centrally as previously described in AML diagnosis [17]. Chromosomal abnormalities and gene mutations of NPM1, FLT3-ITD, CEBPA, DNMT3A, IDH1, and IDH2 were conducted using the previously described methods [18]. We performed the above cytogenetic analyses blinded to both the levels of CALCRL expression and clinical outcome; the clinical characteristics of the patients are summarized in Table 1. All patients provided written informed consent to participate in the study. The Research Ethics Committee of the First Affiliated Hospital, College of Medicine, Zhejiang University, approved this study (reference number: 20201280).

#### 2.6.1. Quantitative Reverse Transcriptase-PCR

Total RNA was isolated from cells using the TRIzol reagent (Cat: 9109, Invitrogen, USA). cDNA was prepared using the reverse transcription kit following the instructions (Thermo, USA). Real-time quantitative PCR (RT-qPCR) was performed in duplicate with the fluorescent DNA binding dye SYBR green (Cat: RR420, Takara, JPN). PCR reactions were performed in a total volume of 25 *μ*l containing 1 *μ*l of 100 ng/*μ*l sample cDNA, 12.5 *μ*l 2× PCR mix, 1 *μ*l 0.5 *μ*M of each primer, and 10.5 *μ*l ddH2O. mRNA levels were normalized to GAPDH. The primers used for quantitative PCR are presented in Table 2.

### 2.7. Western Blot

Total protein was extracted using RIPA buffer (REF: 89900, Sigma-Aldrich, USA) supplemented with protease inhibitor and phosphatase inhibitor cocktail (78420, Thermo Fisher, USA). The protein content was quantified using the BCA protein assay kit (23225, Thermo Fisher). An estimated 40 *μ*g protein was loaded per well on 4–12% SDS-PAGE gel and transferred onto a PVDF membrane (Millipore, Billerica, MA, USA) preactivated with methanol. Membranes were blocked with 5% milk and incubated with antibodies against specific proteins. After overnight, membranes were washed with 1× TBST and incubated with horseradish peroxidase labeled secondary antibodies. The protein bands were visualized using enhanced chemiluminescence reagent (BIO-RAD Gel Dox TM XR+, USA). The antibody of GNAI1 12617-1-AP was purchased from Proteintech (Rosemont, USA), CALCRL (ab84467) was purchased from Abcam (Cambridge, MA, USA), and GAPDH ^#^5174S and the Rabbit (DA1E) mAb IgG secondary antibody were purchased from CST (Danvers, MA) (^#^3900).

#### 2.7.1. RNA-Sequence Data Analysis

The mRNA expression profiles of MV4-11 sensitive cells and MV4-11 resistant strains (MV4-11 R10/R30/R50) were obtained using high-throughput sequencing (RNA-sequence). The sequenced data were obtained from Illumina HiSeq X Ten platforms, and the data were uploaded to NCBI's SRA and access to cite for these SRA data: PRJNA664675. For RNA-seq data, mRNA expression levels were calculated as RPKM (Reads Per Kilo-base per Million reads). We used DE Seq (1.18.0) R package to analyze the differentially expressed mRNAs.

### 2.8. Statistical Analysis

#### 2.8.1. Clinical Data Analysis

Overall survival (OS) was defined as the time from diagnosis to death due to any cause. Event-free survival (EFS) was defined as the time from diagnosis to removal from the study because of the absence of CR, relapse, or death. In the association between CALCRL expression and OS, EFS was estimated by the Kaplan-Meier method and log-rank test. In prognostic analysis of other genes like GNAI1, CALCRL was calculated via online database “GEPIA.”

Experimental analysis, unpaired two-tailed Student's *t*-test, or ANOVA and Chi-square test were performed using IBM SPSS Statistics 20 software or GraphPad Prism 5. *P* < 0.05 was considered statistically significant. *P* < 0.05^*∗*^, *P* < 0.01^*∗∗*^, and *P* < 0.001^*∗∗∗*^.

### 2.9. Hub Gene Analysis

Protein-protein interaction (PPI) network was created by online database “STRING” [19] and visualized by Cytoscape software [20]. Top 10 hub genes were analyzed using cytoHubba APP (22). Biological processes, molecule functions, and cell type analysis were performed using the “Enrichment” online database.

## 3. Results

### 3.1. MV4-11 and MOLM-13 Were the Most Sensitive AML Cell Lines to HHT

**Figure 1:**
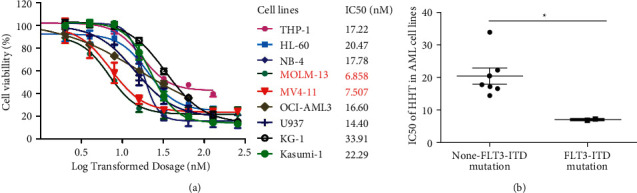
The inhibitory effect of HHT on nine AML cell lines. (a) The IC_50_ values of HHT on nine AML cell lines. (b) The inhibitory effect of HHT on FLT3-ITD mutant cell lines was significantly higher than that in nonmutant ones. ^*∗*^*P* < 0.05.

To explore the cytotoxicity of HHT on AML cells, we detected the antileukemic effect of HHT on nine AML cell lines with different genetic backgrounds via IC_50_ value measurement. The IC_50_ values of MOLM-13 and MV4-11 were 6.858 nM and 7.207 nM, respectively, while other cell lines exceeded 10 nM (Figure 1(a)). The inhibitory effect of HHT on FLT3-ITD mutant cell lines MOLM-13 and MV4-11 was significantly higher than that on the nonmutant cell lines (Figure 1(b)).

### 3.2. Establishment and Verification of the HHT-Resistant Cell Lines

**Figure 2:**
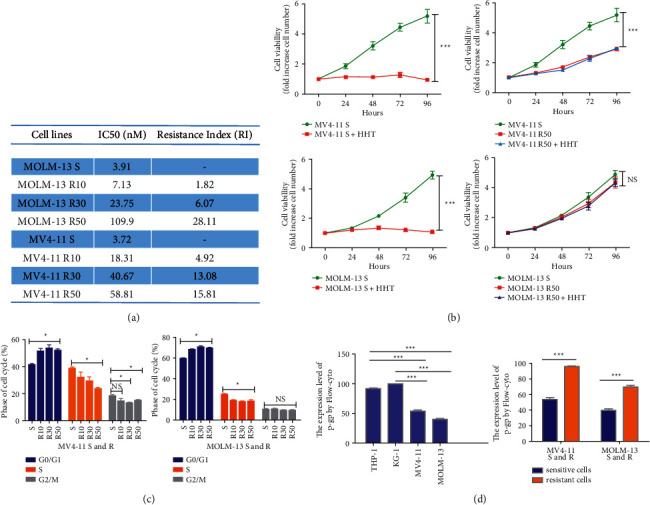
The construction and verification of the HHT-resistant cell lines. (a) The IC_50_ values of HHT-sensitive and HHT-resistant cell lines and the RIs of the constructed HHT-resistant cell lines. (b) The cell growth rate of HHT-resistant cell lines and HHT-sensitive cell liens at 10 nM HHT. (c) The cell cycle of HHT-resistant cell lines and HHT-sensitive cell lines. (d) The protein level of p-gp in HHT-resistant and HHT-sensitive cell lines. NS: not significant. ^*∗*^*P* < 0.05 and ^*∗∗∗*^*P* < 0.001.

As MV4-11 and MOLM-13 were the most sensitive AML cell lines to HHT, these two cell lines were selected to construct HHT-resistant cell lines by exposing them to gradually increasing HHT concentrations for 15 months. Six resistant strains were successfully built, and HHT concentrations were induced at 10 nM, 30 nM, and 50 nM, respectively. The RIs of the three MOLM-13 resistant cell lines (R10, R30, and R50) were 7.13 nM, 23.75 nM, and 109.9 nM, respectively, which were 1.82-fold, 6.07-fold, and 28.11-fold higher than those of the sensitive cells. Contemporarily, the RIs of MV4-11 resistance cell lines (R10, R30, and R50) were 18.31 nM, 40.67 nM, and 58.82 nM, respectively, which were 4.92-fold, 13.08-fold, and 15.81-fold higher than those of sensitive cells (Figure 2(a)). Furthermore, there was no obvious apoptosis of HHT-sensitive cells and HHT-resistant cells under natural culture conditions (Figure S1A). To verify that HHT had no cytotoxic effects on these six cell lines, we evaluated cell growth *in vitro* and *in vivo*. The growth rate of MV4-11 R50 and MOLM-13 R50 was normally or partly inhibited with 10 nM HHT treatment, whereas sensitive cells (MV4-11S and MOLM-13S) were utterly suppressed (Figure 2(b)). In addition, the growth rate of HHT-resistant cells was lower than that of the HHT-sensitive cells (Figure 2(b)). Then we found that G0/G1 phase was delayed, and S phase replication was gradually declined in both MOLM-13 and MV4-11 cells with a progressively increasing HHT RI (Figure 2(c)). Additionally, results from flow cytometry showed that original HHT-resistant strains and constructed resistant cell lines had a higher expression of multidrug resistance protein p-gp (Figure 2(d)).

**Figure 3:**
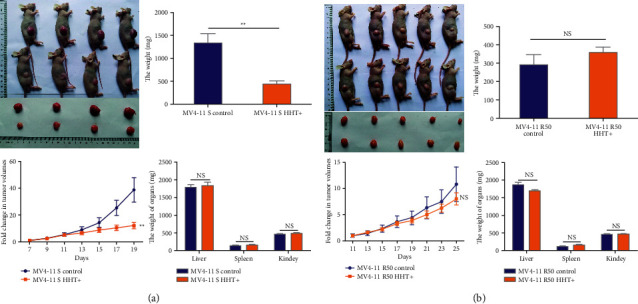
The *in vivo* verification of HHT resistance. (a) The tumor volume, growth rate, and the weight of tumor, liver, spleen, and kidney of mice in HHT-sensitive mice model. (b) The tumor volume, growth rate, and the weight of tumor, liver, spleen, and kidney of mice in HHT-sensitive mice model. NS: not significant. ^∗∗^*P* < 0.01.

Next, we used the nude mouse subcutaneous tumor-bearing model to observe the drug resistance of HHT-resistant strains *in vivo*. Subcutaneous tumor mass in mice is mainly infiltrated by leukemia cells (Figure S1B). The animal experimental results also showed that tumor volume, growth rate, and weight were not significantly decreased after HHT treatment in MV4-11 R50 mice, while they were significantly decreased in MV4-11 sensitive mice (Figures 3(a)-3(b)). After HHT treatment, tumor volume was reduced more significantly in the MV4-11 sensitive group than in the resistant group (Figure S1C). These results revealed six HHT-resistant cell lines with different RIs were successfully established.

### 3.3. Differentially Expressed Genes (DEGs) and Pathways between Sensitive and Resistant Strains

**Figure 4:**
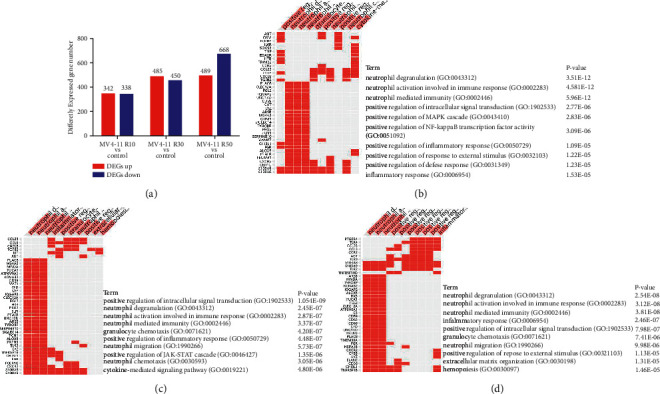
Differential expression genes (DEGs) and pathways between sensitive and resistant strains. (a) There were 342, 485, and 489 upregulated genes and 338, 450, and 668 downregulated genes in the three sets MV4-11 R10 versus MV4-11 (S), MV4-11 R30 versus MV4-11 (S), and MV4-11 R50 versus MV4-11 (S). (b) BP analysis among MV4-11 R10 and MV4-11. (c) BP analysis among MV4-11 R30 and MV4-11. (d) BP analysis among MV4-11 R50 and MV4-11.

As the HHT-resistant cell lines were successfully established, we aimed to explore the dynamic changes in gene expression with gradually increasing HHT RIs. Gene expression profiles between different RIs of HHT-sensitive and HHT-resistant cells were analyzed by RNA-sequence. There were 342, 485, and 489 upregulated genes and 338, 450, and 668 downregulated genes in the three sets MV4-11 R10 versus MV4-11 S, MV4-11 R30 versus MV4-11 S, and MV4-11 R50 versus MV4-11 S, respectively (Figure 4(a)). The RT-qPCR results from three upregulated genes and three downregulated genes verified the reliability of RNA-sequence (Figure S2). Further intersection analyses showed that 227 genes were all upregulated, and 243 were downregulated in three MV4-11 resistant strains by Venn methods (Figure S3).

To better understand the biological functions of these DEGs, we performed enrichment analysis. Upregulated genes in resistant groups took part in several stable pathways related to the immune activity, such as neutrophil activation involved in immune response and neutrophil-mediated immunity (Figures 4(b)–4(d)). The downregulated genes participated in several metabolic and biosynthetic processes, and the differences increased as the resistance index increased (Figures S4A–S4C).

### 3.4. Key Regulated DEGs in HHT-Resistant Cells

**Figure 5:**
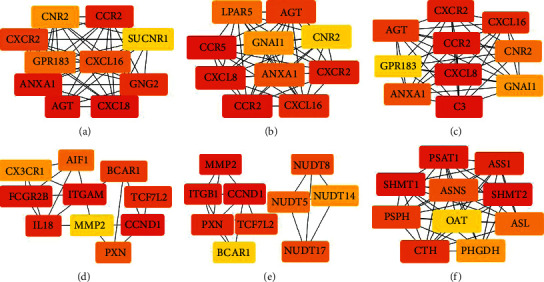
The hub genes, respectively, in both upregulated and downregulated differential genes among three sets. (a) The top 10 hub genes among upregulated genes in MV4-11 R10 versus MV4-11 sets. (b) The top 10 hub genes among upregulated genes in MV4-11 R30 versus MV4-11 sets. (c) The top 10 hub genes among upregulated genes in MV4-11 R50 versus MV4-11 sets. (d) The top 10 hub genes among downregulated genes in MV4-11 R10 versus MV4-11 sets. (e) The top 10 hub genes among downregulated genes in MV4-11 R30 versus MV4-11 sets. (f) The top 10 hub genes among downregulated genes in MV4-11 R50 versus MV4-11 sets.

To explore the critical proteins in HHT resistance, PPI nodes from the online database “STRING” were obtained to discover the interactions between proteins. In addition, the top 10 hub genes were analyzed using cytoHubba software from both upregulated and downregulated DEGs among three sets; the top 10 hub genes among upregulated DEGs are shown in Figures 5(a)–5(c) with increasing RI, and the top 10 hub genes among downregulated DEGs are shown in Figures 5(d)–5(f). From biological function (BP) analysis, upregulated hub genes in MV4-11 resistant strains took part in dendritic cell chemotaxis; G-protein coupled receptor signaling pathway, coupled to cyclic nucleotide second messenger; positive regulation of T-helper 1 type immune response; and so forth (Figure S5). In those processes, the biological processes “G-protein coupled receptor (GPR) signaling pathway, coupled to cyclic nucleotide second messenger” were significantly upregulated as the RI increased (Figure S5). These results indicated that immune biology process, especially GPR signaling, may mediate HHT resistance. The biological processes of downregulated genes included a variety of biological processes, while the metabolic and biosynthetic processes were not stable as the resistance index decreased (Figure S6).

### 3.5. CALCRL and GNAI1 May Mediate HHT Resistance and Have Clinical Significance in AML

**Figure 6:**
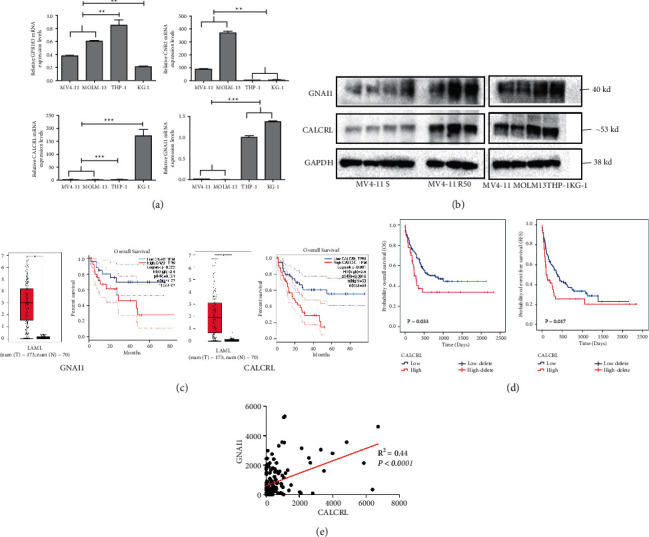
Verification of the hub genes (CALCRL and GNAI1) and the prognostic significance of CALCRL and GNAI1 for AML. (a) The mRNA level of GPR183, CNR2, CALCRL, and GNAI1 in four AML cell lines by RT-qPCR. (b) The protein level of CALCRL and GNAI1 in four AML cell lines and the mice tumor mass from HHT-sensitive and HHT-resistant mice groups. (c) The expression level and OS of GNAI1 and CALCRL in online database GEPIA in AML. (d) The OS and EFS of CALCRL in our cohorts. (e) The correlation of GNAI1 and CALCRL through correlation analysis. *P* < 0.01^*∗∗*^ and *P* < 0.001^*∗∗∗*^.

Given that immune biological processes, especially GPR signaling, may mediate HHT resistance, we further focused on four upregulated DEGs (GPR183, CNR2, CALCR, and GNAI1), which belong to the G-protein family. First, the mRNA expression levels of these genes were detected in the HHT innate resistant cell lines THP-1 and KG-1 and sensitive cell lines MV4-11 and MOLM-13. Only CALCRL and GNAI1 had higher expression levels in innate resistant cells THP-1and KG-1 (Figure 6(a)). Contemporarily, the protein levels of CALCRL and GNAI1 were also higher in innate resistant cells and mice tumor (Figure 6(b)). In addition, compared to the MV4-11 sensitive mice group, the MV4-11 R50 mice had higher protein levels of CALCRL and GNAI1 (Figure 6(b)). Then, we explored the clinical significance of CARCRL and GNAI1 in AML. First, higher expression levels of CALCRL and GNAI1 were found in AML cells than in the normal cells (Figure 6(c)). Moreover, patients with high GNAI1 expression tended to have a poor prognosis according to the public database GEPIA (Figure 6(c)). Patients with high CALCRL expression had also worse OS from GEPIA (Figure 6(c)). We also verified the clinical significance of CALCRL by our database, and the results showed that AML patients with high CALCRL expression had poor OS and EFS (Figure 6(d)). Interestingly, CALCRL and GNAI1 had significant positive correlation by analyzing the TCGA database (Figure 6(e)). These results indicated that CALCRL and GNAI1 were highly expressed in HHT-resistant cells and may mediate HHT resistance and might have interaction.

## 4. Discussion

With the widespread use of HHT in AML treatment, drug resistance has become a great challenge, and few attempts have been made to explore the resistance mechanisms. In this study, we successfully constructed six HHT-resistant cell lines in two HHT-sensitive AML cell lines with different RIs. The growth rate of HHT-resistant cell lines was decreased and accompanied by a delay in the cell cycle and an increased p-gp (CD243) expression. GPRs mediate immune activity, for molecular mechanisms may play an essential role in HHT resistance. The GPR signaling, especially two GPRs, CALCRL and GNAI1, may mediate HHT resistance and have prognostic significance in AML.

Drug-resistant cell lines can be used to study biological resistance changes. In this study, we constructed different RIs of cell lines to observe the resistance characteristics and mechanism dynamically. Recent studies have shown that the cell cycle plays a critical role in chemotherapeutic resistance in cancer. When cancer cells exhibit Palbociclib resistance, cells are delayed in the G1 phase [21]. Further studies revealed that cancer cells with delayed G1 phase could affect intracellular platinum concentrations and protect them from chemotherapy-induced damage [22]. Another study found a lower percentage of S phase in paclitaxel-resistant cell lines of ovarian carcinoma [23]. These studies were consistent with our results in HHT-resistant cell lines, in which G0/*G*1 phase was delayed and S phase was decreased in HHT-resistant cells.

The increase of p-gp expression in resistant cells was another crucial difference between resistant and sensitive cells. Overexpression of p-gp is considered the primary cause of multidrug resistance (MDR) in AML (26, 27). p-gp functions as an ATP-dependent membrane protein and is involved in the absorption, distribution, and excretion of drugs, xenobiotics, and endogenous compounds [24]. These results indicate that cell cycle delay reduces cell growth rate, and increasing p-gp expression may induce HHT resistance in AML cells.

After KEGG and hub gene analyses, we found that immune activity may play an important role in HHT resistance, and top 10 upregulated hub genes were focused on GPRs. GPRs can regulate many fundamental biological processes, such as cardiovascular functions, immune responses, and hormones [25]. Dysregulated expression and aberrant function of GPRs have been linked to many human diseases including cancer [26]. Cancer immunotherapy targeting GPRs is a promising treatment strategy [27]. In this study, the critical DEGs after HHT resistance belonged to GPRs, indicating that immune regulation by GRPs may play an essential role in HHT resistance. We focused on upregulated DEGs which belonged to GPRs and found that two upregulated DEGs, CALCRL (31, 32) and GNAI1 (33, 34), were highly expressed in primary HHT-resistant cell lines and newly constructed HHT-resistant cell lines. These indicated that CALCRL and GNAI1 may mediate HHT resistance in AML cells. GNAI1 has been confirmed as an oncogene and therapeutic target in serous ovarian cancer [28] and hepatocellular carcinoma [29]. However, its role in AML remains poorly understood. CALCRL is a potential therapeutic target for AML [30]. Another study found that CALCRL increased chemotherapy resistance (Ara-c and daunorubicin) and stem cell properties in AML (32). These confirmed the important role of the two genes in cancer therapy including AML. From UniProt database, we knew that CALCRL was localized in the cell membrane, but GNAI1 was widely localized in various parts of the cell. As we found that they had significant positive correlation, whether they interact to mediate HHT resistance in AML is unclear. So, in this study, we did not further explore the exact role and mechanism of CALCRL and GNAI1 in regulating HHT resistance in AML; we also did not reveal the interaction between CALCRL and GNAI1; this requires future detailed studies. Further research using targeting knocked down or overexpression methods is needed.

## 5. Conclusion

In summary, we successfully constructed six HHT-resistant AML cell lines with different RIs; these will be specific cell tools to study HHT resistance. The cell cycle delay and increase in p-gp expression may be the biological reasons for HHT resistance. At the transcriptional level, we found that GPRs, especially CALCRL and GNAI1, may mediate HHT resistance. There are some limitations in this study; although we found that GPRs may mediate HHT, we did not study how to mediate HHT resistance, and we need to further explore how CALCRL and GNAI1 regulate HHT resistance in AML and even chemotherapy resistance in AML. The interrelationship and role of CALCRL and GNAI1 also require further research in the future. We only explored the possible mechanism of HHT resistance in AML at the transcriptional level. Further, we need to use the constructed HHT-resistant cell lines to comprehensively study the HHT resistance mechanism at the metabolic and proteomic levels and to explore whether each omics level there is a cross.

## Figures and Tables

**Table 1:** Comparison of the CALCRL expression characteristics of AML patients between low group and high group.

Variables	CALCRL expression		*P* value
	Low group	High group	
Number, *n* (%)	130 (67)	43 (33)	
Age, median (range)	51.5 (18,80)	51 (14,69)	0.647
Female, *n* (%)	55 (42.3)	21 (48.8)	0.482
WBC^1^, median (range)	13.4 (0.2, 453.2)	37.5 (0.9, 214.0)	0.235
HB^2^, median (range)	81.0 (33.5, 154.0)	74.0 (53.0, 134)	0.090
PLT^3^, median (range)	42 (2, 776)	44 (4, 778)	0.736
BM^4^ blast, median (range) %	69 (17.5, 96.65)	67.2 (23, 95)	0.645
FAB^5^ type			0.447
M0	8 (6.2)	3 (7.0)	
M1	13 (10.0)	2 (4.7)	
M2	53 (40.8)	24 (55.8)	
M4	6 (4.6)	3 (7.0)	
M5	40 (30.8)	10 (23.3)	
M6	4 (0.1)	0 (0)	
Karyotype risk^6,^*n* (%)			1.000
Favorable	5 (3.8)	1 (2.3)	
Intermediate	105 (80.8)	38 (88.4)	
Unfavorable	6 (4.6)	2 (4.7)	
Gene mutation			
FLT3-ITD	18 (16.2)	26 (23.4)	0.002
NPM1	24 (21.6)	38 (34.2)	0.693
CEBPA^DM7^	19 (17.1)	13 (11.7)	1.000
IDH1	5 (4.5)	14 (12.6)	0.375
IDH2	9 (8.1)	12 (10.8)	0.778
DNMT3A	7 (6.3)	16 (14.4)	0.775

^1^WBC, white blood cell; ^2^HB, hemoglobin; ^3^PLT, platelet counts; ^4^BM, bone marrow; ^5^FAB, French-American-British classification systems; ^6^karyotype risk. Favorable subgroup comprised *t* (8, 21)/AML1-ETO, inv16 or *t* (16, 16)(p13.1; q22)/CBFb-MYH11,CEPRA^DM^, NPM1 without FLT3-ITD or FLT3-ITD^low^. Adverse group consisted of *t* (8, 16), inv (3)/*t* (3, 3), −5, −7, del (5q), del (7p), 11q23, and complex translocations. Intermediate subtype contained cytogenetically normal and AML with other cytogenetic abnormalities. ^7^DM, double-allele.

**Table 2:** The sequence of the primers.

Gene name	Sense primer	Antisense primer
CALCRL	5′-TCCTGAGGACTCAATTCAGTTGG-3′	5′-CTGTTGCAGTAAACGCCTTCT-3′
CNR2	5′-AGCCCTCATACCTGTTCATTGG-3′	5′-GTGAAGGTCATAGTCACGCTG-3′
GPR183	5′-ACTGGAGAATCGGAGATGCCT-3′	5′-AATGAAGCGGTCAATACTCAGG-3′
GNAI1	5′-TTAGGGCTATGGGGAGGTTGA-3′	5′-GGTACTCTCGGGATCTGTTGAAA-3′
GAPDH	5′-GGAGCGAGATCCCTCCAAAAT-3′	5′-GGCTGTTGTCATACTTCTCATGG-3′
GATM	5′-CTAAGGGGTCCCATTCGTTGT-3′	5′-CACTACATCGGATCTCGGCTT-3′
P4HTM	5′-ATGGATGATGAGCCGACACTC-3′	5′-TCACCGACAGGGATCACTTCA-3′
SLC22A16	5′-TCACCGCAGTGCTTTTCCA-3′	5′-GGAATAAGAGGGAGAACACATC-3′
CHI3L1	5′-GTGAAGGCGTCTCAAACAGG-3′	5′-GAAGCGGTCAAGGGCATCT-3′
CLEC12 A	5′-TAGCCACCAAATTATGTCGTGAG-3′	5′-GCTGTCCTTATGCCAAATCCATC-3′
GATA2	5′-ACTGACGGAGAGCATGAAGAT-3′	5′-CCGGCACATAGGAGGGGTA-3′

## Data Availability

The datasets used and analyzed during this study are available from the corresponding author upon reasonable request.

## Abbreviations

HHT: Homoharringtonine
HAA: HHT + aclarubicin + cytarabine
AML: Acute myeloid leukemia
RI: Resistance index
p-gp: P-glycoprotein (CD243)
KEGG: Kyoto Encyclopedia of Genes and Genomes
GO: Gene Ontology
GPR: G-protein coupled receptor
CALCRL: Calcitonin Receptor-Like
GNAI1: G Protein Subunit Alpha I1
CML: Chronic myeloid leukemia
MDS: Myelodysplastic syndrome
CR: Complete remission
EFS: Event-free survival
DA: Daunorubicin plus cytarabine
IA: Idarubicin plus cytarabine
IC_50_: Half-maximal inhibitory concentration
APL: Acute promyelocytic leukemia
PI: Propidium
RT-qPCR: Real-time quantitative PCR
OS: Overall survival
DEGs: Differentially expressed genes
MDR: Multidrug resistance.

## Supplementary Materials

Supplementary Materials Figure S1: the phenotype of HHT-resistant and HHT-sensitive strains. A: the apoptosis of HHT-resistant cell lines and HHT-sensitive cell lines without HHT cultivate. B: the HE stains of mice tumor mass between HHT-sensitive and HHT-resistant groups. C: the fold change of tumor volume after HHT treatment. NS: not significant; ^*∗∗∗*^*P* < 0.001. Figure S2: the verification of DEGs in three MV4-11 resistant cell lines compared with MV4-11 sensitive cell lines. A: fold change of upregulated gene expression in three MV4-11 resistant cell lines via RT-qPCR. B: fold change of downregulated gene expression in three MV4-11 resistant cell lines via RT-qPCR. Figure S3: intersection between MV4-11 R10 versus MV4-11, MV4-11 R30 versus MV4-11, and MV4-11 R50 versus MV4-11. A: in these three sets, there were 227 upregulated genes. B: in these three sets, there were 243 downregulated genes. Figure S4: BP about downregulated genes among MV4-11 resistant strains and sensitive ones. A: BP analysis among MV4-11 R10 and MV4-11. B: BP analysis among MV4-11 R30 and MV4-11. C: BP analysis among MV4-11 R50 and MV4-11. Figure S5: BP analysis of upregulated hub genes in MV4-11 resistant strains. A: BP analysis of upregulated hub genes between MV4-11 R10 resistant strains and MV4-11 sensitive ones. B: BP analysis of upregulated hub genes between MV4-11 R30 resistant strains and MV4-11 sensitive ones. C: BP analysis of upregulated hub genes between MV4-11 R50 resistant strains and MV4-11 sensitive ones. Figure S6: BP analysis of downregulated hub genes in MV4-11 resistant strains. A: BP analysis of downregulated hub genes between MV4-11 R10 resistant strains and MV4-11 sensitive ones. B: BP analysis of downregulated hub genes between MV4-11 R30 resistant strains and MV4-11 sensitive ones. C: BP analysis of downregulated hub genes between MV4-11 R50 resistant strains and MV4-11 sensitive ones.
