# Supplementary material for: Characterization of the Newly Established Homoharringtonine- (HHT-) Resistant Cell Lines and Mechanisms of Resistance
Source: J Oncol. 2022 Aug 30;2022:2813938. doi: 10.1155/2022/2813938 (PMC9448541; doi:10.1155/2022/2813938)
Supplement: Supplementary Materials — Figure S1: the phenotype of HHT-resistant and HHT-sensitive strains. A: the apoptosis of HHT-resistant cell lines and HHT-sensitive cell lines without HHT cultivate. B: the HE stains of mice tumor mass between HHT-sensitive and HHT-resistant groups. C: the fold change of tumor volume after HHT treatment. NS: not significant; ∗∗∗P < 0.001. Figure S2: the verification of DEGs in three MV4-11 resistant cell lines compared with MV4-11 sensitive cell lines. A: fold change of upregulated gene expression in three MV4-11 resistant cell lines via RT-qPCR. B: fold change of downregulated gene expression in three MV4-11 resistant cell lines via RT-qPCR. Figure S3: intersection between MV4-11 R10 versus MV4-11, MV4-11 R30 versus MV4-11, and MV4-11 R50 versus MV4-11. A: in these three sets, there were 227 upregulated genes. B: in these three sets, there were 243 downregulated genes. Figure S4: BP about downregulated genes among MV4-11 resistant strains and sensitive ones. A: BP analysis among MV4-11 R10 and MV4-11. B: BP analysis among MV4-11 R30 and MV4-11. C: BP analysis among MV4-11 R50 and MV4-11. Figure S5: BP analysis of upregulated hub genes in MV4-11 resistant strains. A: BP analysis of upregulated hub genes between MV4-11 R10 resistant strains and MV4-11 sensitive ones. B: BP analysis of upregulated hub genes between MV4-11 R30 resistant strains and MV4-11 sensitive ones. C: BP analysis of upregulated hub genes between MV4-11 R50 resistant strains and MV4-11 sensitive ones. Figure S6: BP analysis of downregulated hub genes in MV4-11 resistant strains. A: BP analysis of downregulated hub genes between MV4-11 R10 resistant strains and MV4-11 sensitive ones. B: BP analysis of downregulated hub genes between MV4-11 R30 resistant strains and MV4-11 sensitive ones. C: BP analysis of downregulated hub genes between MV4-11 R50 resistant strains and MV4-11 sensitive ones. [file 2813938.f1.pdf]

Supplementary Figure legends

Figure.S1

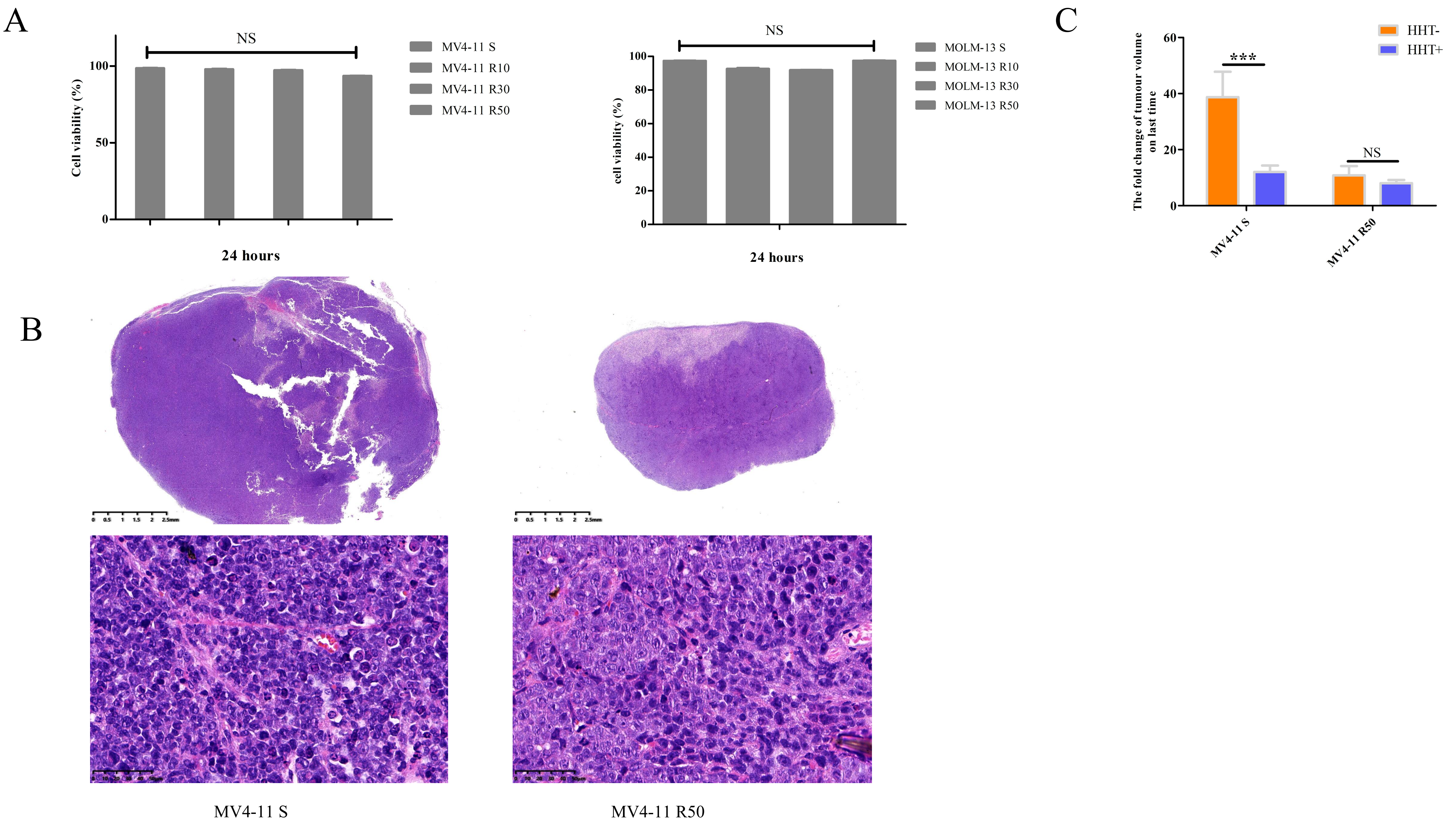
A
NS
Cell viability (%)
100
50
0
24 hours
MV4-11 S
MV4-11 R10
MV4-11 R30
MV4-11 R50
NS
cell viability (%)
100
80
60
40
20
0
24 hours
MOLM-13 S
MOLM-13 R10
MOLM-13 R30
MOLM-13 R50
C
HHT-
HHT+
The fold change of tumour volume on last time
60
40
20
0
***
NS
MV4-11 S
MV4-11 R50
B
MV4-11 S
MV4-11 R50

Figure.S2

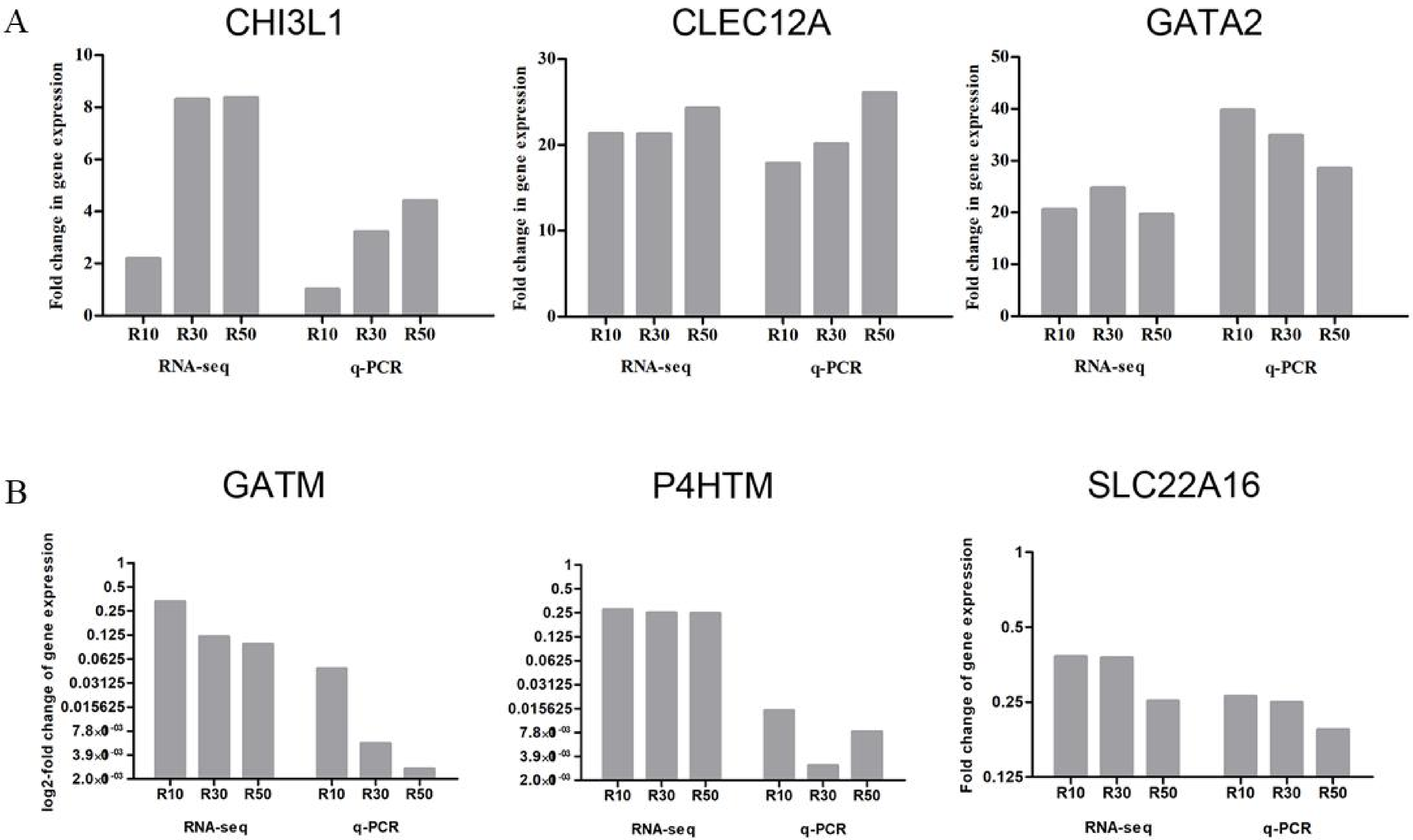
A
CHI3L1
CLEC12A
GATA2
Fold change in gene expression
0
2
4
6
8
10
0
10
20
30
0
10
20
30
40
50
R10 R30 R50 R10 R30 R50
RNA-seq
q-PCR
B
GATM
P4HTM
SLC22A16
log2-fold change of gene expression
Fold change of gene expression
1
0.5
0.25
0.125
0.0625
0.03125
0.015625
7.8×0⁻⁰³
3.9×0⁻⁰³
2.0×0⁻⁰³
0.125
R10 R30 R50 R10 R30 R50
RNA-seq
q-PCR

Figure.S3

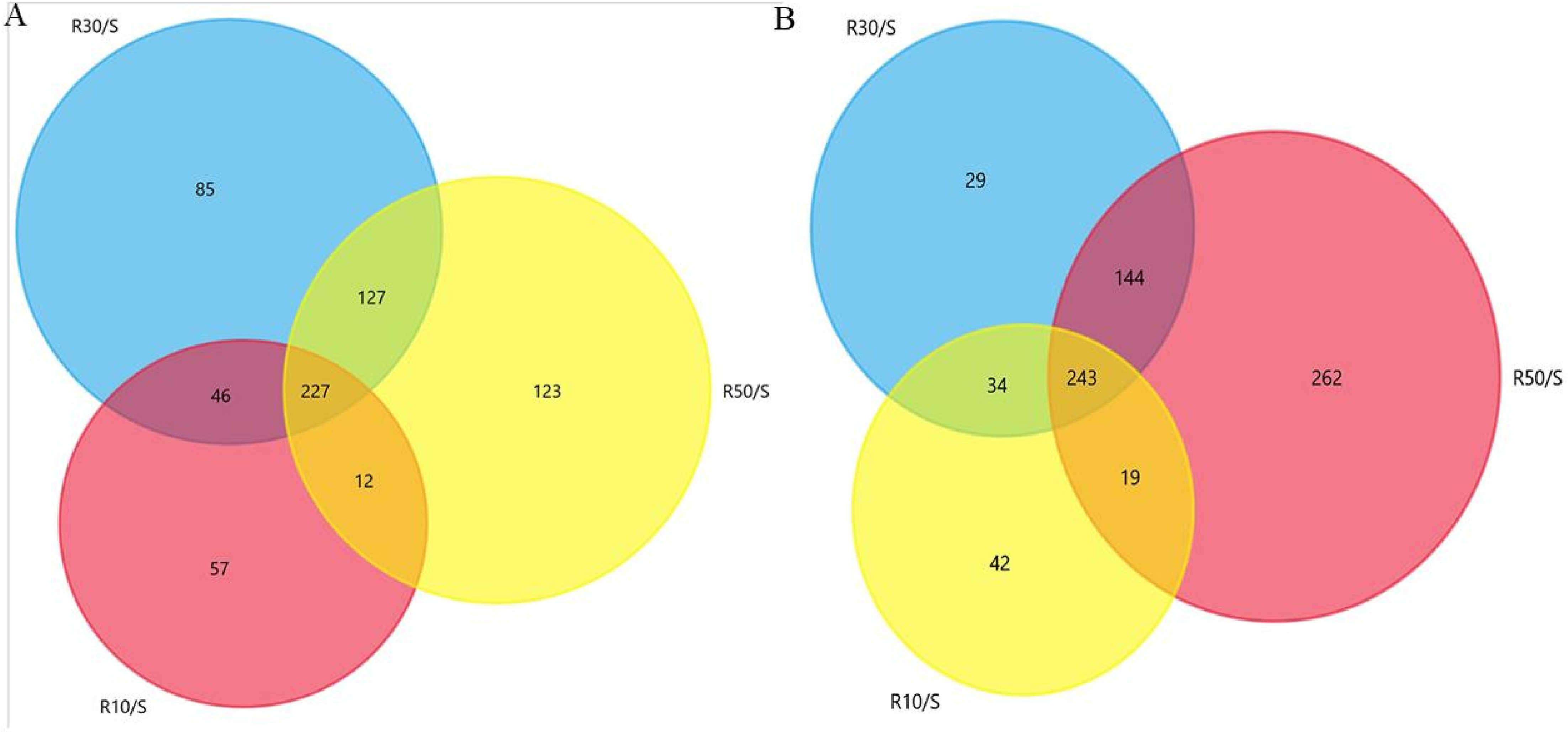
A
R30/S
85
127
46
227
123
R50/S
12
57
R10/S
B
R30/S
29
144
34
243
262
R50/S
19
42
R10/S

Figure.S4

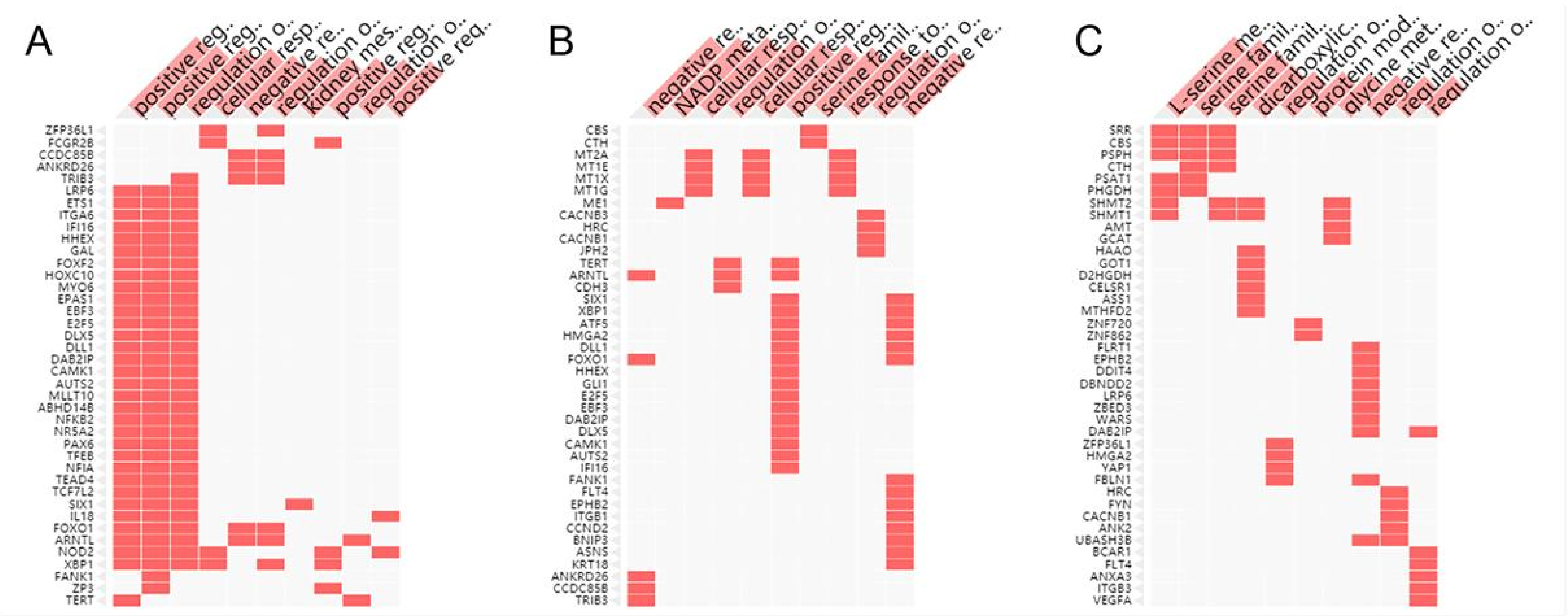

Term	P-value
positive regulation of transcription from RNA polymerase II promoter (GO:0045944)	7.93E-06
positive regulation of transcription, DNA-templated (GO:0045893)	3.51E-05
regulation of transcription from RNA polymerase II promoter (GO:0006357)	4.31E-05
cellular response to peptide (GO:1901653)	6.08E-05
negative regulation of fat cell differentiation (GO:0045599)	1.85E-04
regulation of fat cell differentiation (GO:0045598)	2.13E-04
kidney mesenchyme development (GO:0072074)	2.52E-04
positive regulation of immune response (GO:0050778)	2.91E-04
regulation of hair cycle (GO:0042634)	3.73E-04
positive regulation of type 2 immune response (GO:0002830)	5.26E-04

Term	P-value
negative regulation of fat cell differentiation (GO:0045599)	6.88E-04
NADP metabolic process (GO:0006739)	7.49E-04
cellular response to zinc ion (GO:0071294)	7.49E-04
regulation of hair cycle (GO:0042634)	8.59E-04
cellular response to copper ion (GO:0071280)	0.001117
positive regulation of transcription from RNA polymerase II promoter (GO:0045944)	0.001671
serine family amino acid biosynthetic process (GO:0009070)	0.002140
response to copper ion (GO:0046688)	0.002199
regulation of calcium ion transmembrane transporter activity (GO:1901019)	0.002944
negative regulation of apoptotic process (GO:0043066)	0.003428

Term	P-value
L-serine metabolic process (GO:0006563)	1.32E-08
serine family amino acid biosynthetic process (GO:0009070)	3.08E-08
serine family amino acid metabolic process (GO:0009069)	6.52E-07
dicarboxylic acid metabolic process (GO:0043648)	2.31E-05
regulation of stem cell proliferation (GO:0072091)	1.32E-04
protein modification by small protein conjugation or removal (GO:0070647)	1.36E-04
glycine metabolic process (GO:0006544)	2.21E-04
negative regulation of protein phosphorylation (GO:0001933)	3.23E-04
regulation of calcium ion transmembrane transport (GO:1903169)	4.18E-04
regulation of endothelial cell migration (GO:0010594)	4.64E-04

Figure.S5

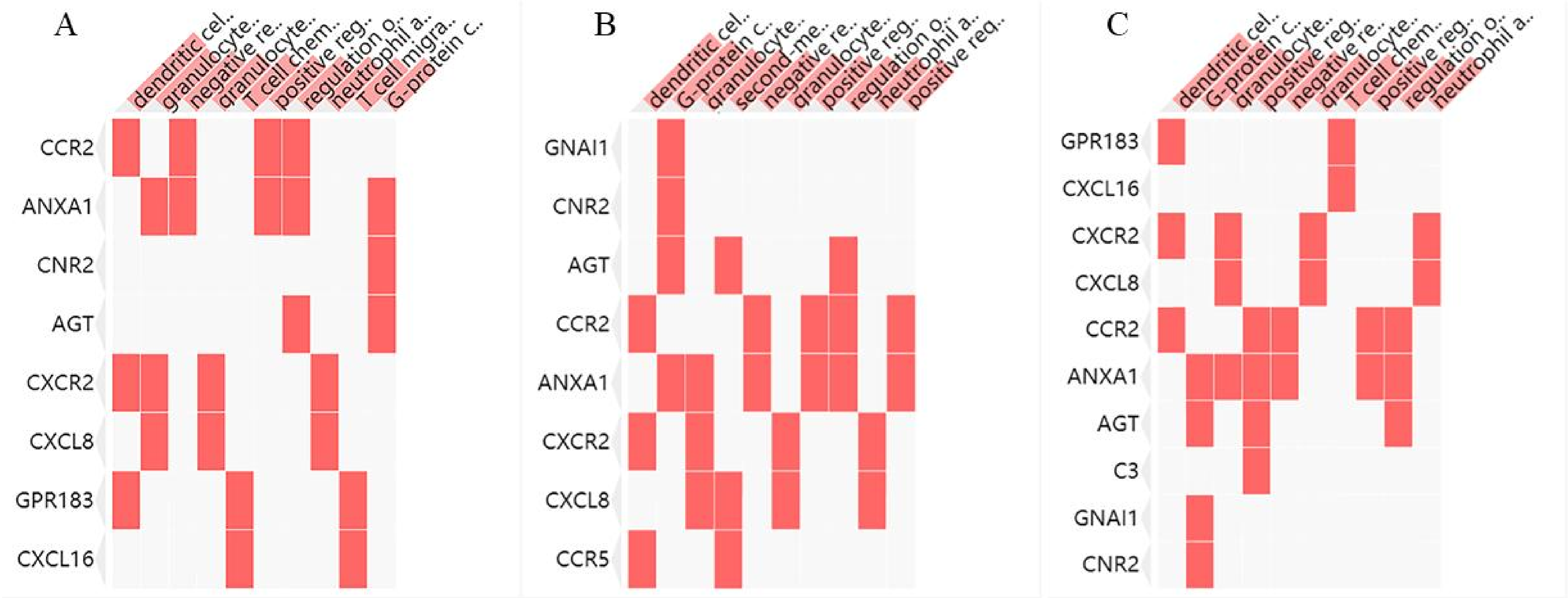

Term	P-value
dendritic cell chemotaxis (GO:0002407)	5.02E-08
granulocyte chemotaxis (GO:0071621)	2.46E-06
negative regulation of type 2 immune response (GO:0002829)	4.72E-06
granulocyte activation (GO:0036230)	8.08E-06
T cell chemotaxis (GO:0010818)	1.01E-05
positive regulation of T-helper 1 type immune response (GO:0002827)	1.48E-05
regulation of cytokine production (GO:0001817)	1.79E-05
neutrophil activation (GO:0042119)	2.04E-05
T cell migration (GO:0072678)	2.69E-05
G-protein coupled receptor signaling pathway, coupled to cyclic nucleotide second messenger (GO:0007187)	2.77E-05

Term	P-value
dendritic cell chemotaxis (GO:0002407)	5.02E-08
G-protein coupled receptor signaling pathway, coupled to cyclic nucleotide second messenger (GO:0007187)	2.97E-07
granulocyte chemotaxis (GO:0071621)	2.46E-06
second-messenger-mediated signaling (GO:0019932)	3.35E-06
negative regulation of type 2 immune response (GO:0002829)	4.72E-06
granulocyte activation (GO:0036230)	8.08E-06
positive regulation of T-helper 1 type immune response (GO:0002827)	1.48E-05
regulation of cytokine production (GO:0001817)	1.79E-05
neutrophil activation (GO:0042119)	2.04E-05
positive regulation of interleukin-2 production (GO:0032743)	4.25E-05

Term	P-value
dendritic cell chemotaxis (GO:0002407)	5.02E-08
G-protein coupled receptor signaling pathway, coupled to cyclic nucleotide second messenger (GO:0007187)	2.97E-07
granulocyte chemotaxis (GO:0071621)	2.46E-06
positive regulation of cytokine production (GO:0001819)	2.84E-06
negative regulation of type 2 immune response (GO:0002829)	4.72E-06
granulocyte activation (GO:0036230)	8.08E-06
T cell chemotaxis (GO:0010818)	1.01E-05
positive regulation of T-helper 1 type immune response (GO:0002827)	1.48E-05
regulation of cytokine production (GO:0001817)	1.79E-05
neutrophil activation (GO:0042119)	2.04E-05

Figure.S6

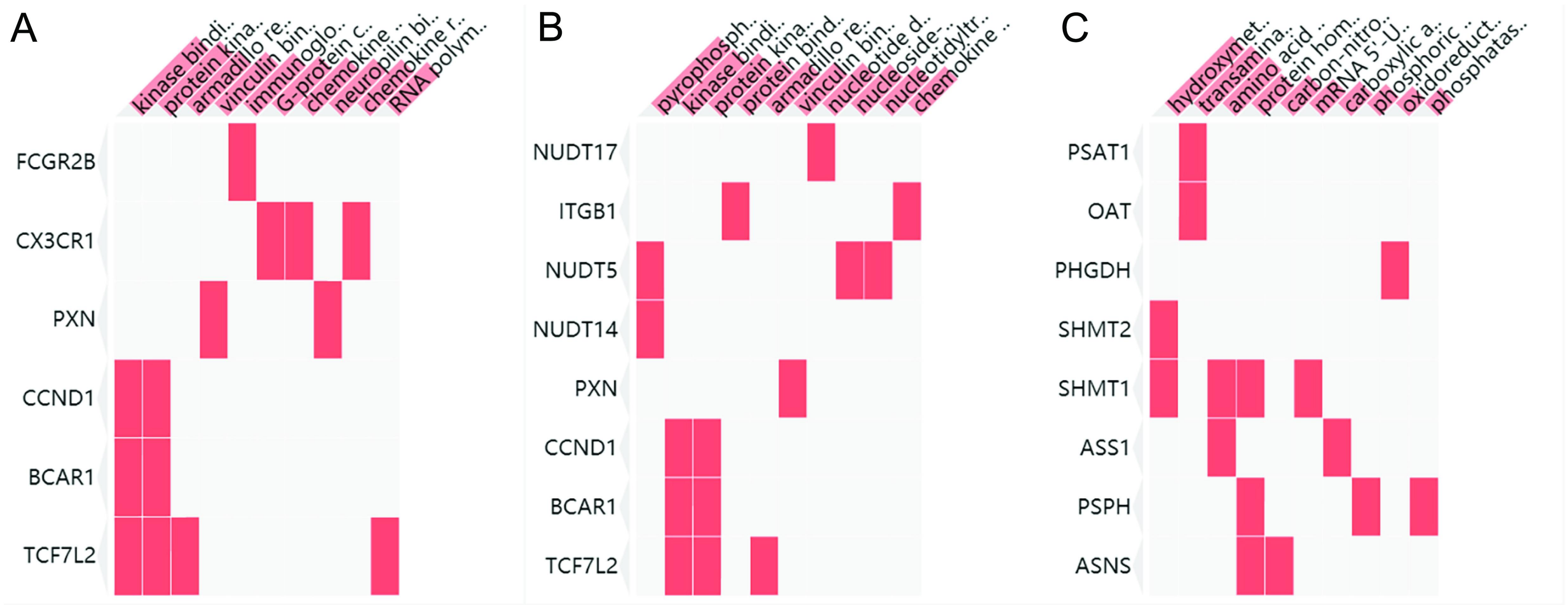

Term	P-value
kinase binding (GO:0019900)	9.75E-04
protein kinase binding (GO:0019901)	0.001588
armadillo repeat domain binding (GO:0070016)	0.004492
vinculin binding (GO:0017166)	0.004990
immunoglobulin binding (GO:0019865)	0.005488
G-protein coupled chemoattractant receptor activity (GO:0001637)	0.007476
chemokine binding (GO:0019956)	0.008469
neuropilin binding (GO:0038191)	0.009462
chemokine receptor activity (GO:0004950)	0.010453
RNA polymerase II repressing transcription factor binding (GO:0001103)	0.013915

Term	P-value
pyrophosphatase activity (GO:0016462)	1.83E-04
kinase binding (GO:0019900)	9.75E-04
protein kinase binding (GO:0019901)	0.001588
protein binding involved in cell-matrix adhesion (GO:0098634)	0.003495
armadillo repeat domain binding (GO:0070016)	0.004492
vinculin binding (GO:0017166)	0.004990
nucleotide diphosphatase activity (GO:0004551)	0.005488
nucleoside-diphosphatase activity (GO:0017110)	0.006482
nucleotidyltransferase activity (GO:0016779)	0.007973
chemokine binding (GO:0019956)	0.008469

Term	P-value
hydroxymethyl-, formyl- and related transferase activity (GO:0016742)	3.37E-06
transaminase activity (GO:0008483)	2.04E-05
amino acid binding (GO:0016597)	1.65E-04
protein homodimerization activity (GO:0042803)	0.003671
carbon-nitrogen ligase activity, with glutamine as amido-N-donor (GO:0016884)	0.004990
mRNA 5'-UTR binding (GO:0048027)	0.010949
carboxylic acid binding (GO:0031406)	0.016874
phosphoric ester hydrolase activity (GO:0042578)	0.027656
oxidoreductase activity, acting on the CH-OH group of donors, NAD or NADP as acceptor (GO:0016616)	0.043148
phosphatase activity (GO:0016791)	0.050811

Supplementary materials

Cell cycle

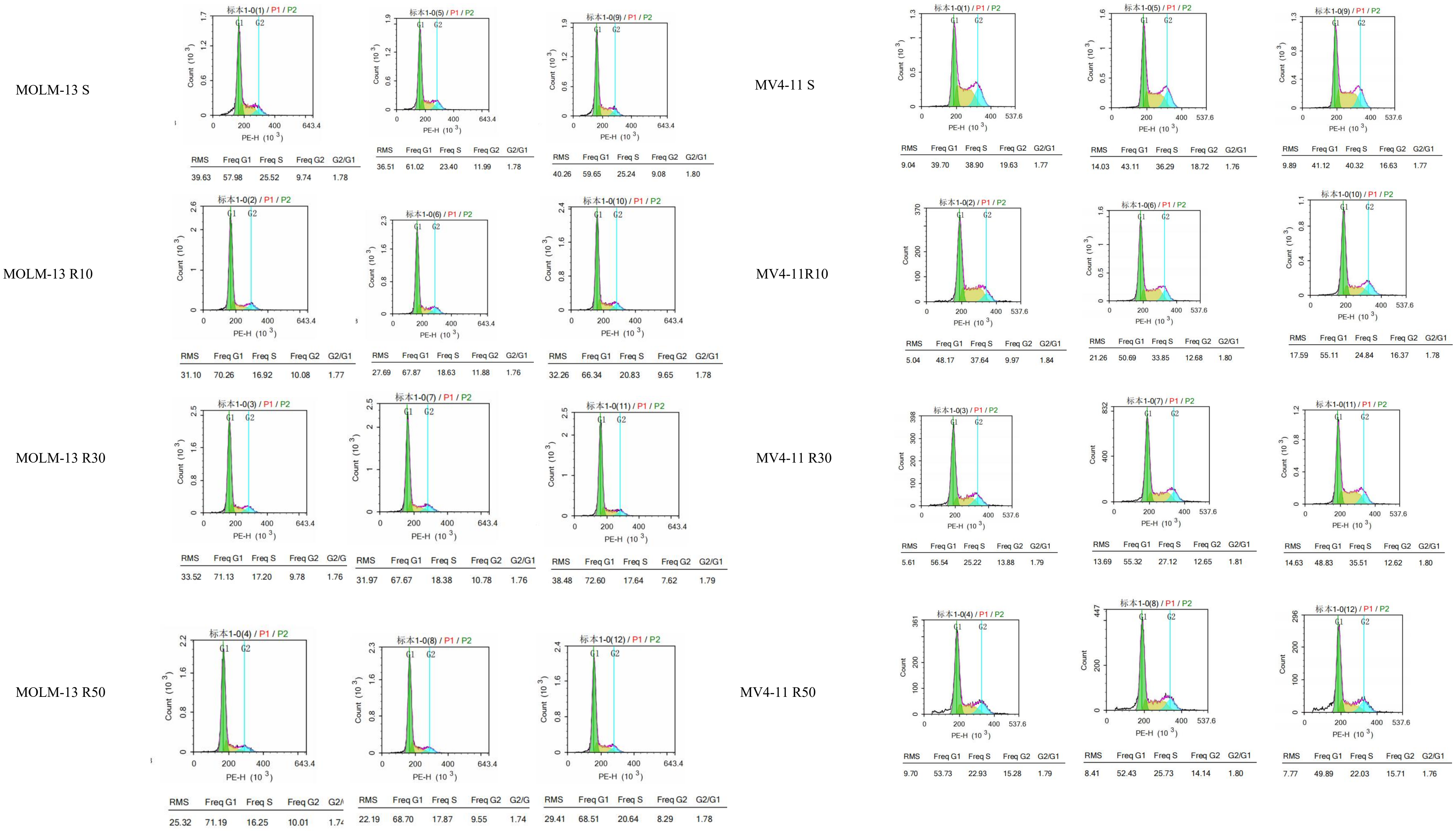
MOLM-13 S
标本1-0(1) / P1 / P2
RMS Freq G1 Freq S Freq G2 G2/G1
39.63 57.98 25.52 9.74 1.78
标本1-0(5) / P1 / P2
RMS Freq G1 Freq S Freq G2 G2/G1
36.51 61.02 23.40 11.99 1.78
标本1-0(9) / P1 / P2
RMS Freq G1 Freq S Freq G2 G2/G1
40.26 59.65 25.24 9.08 1.80
MOLM-13 R10
标本1-0(2) / P1 / P2
RMS Freq G1 Freq S Freq G2 G2/G1
31.10 70.26 16.92 10.08 1.77
标本1-0(6) / P1 / P2
RMS Freq G1 Freq S Freq G2 G2/G1
27.69 67.87 18.63 11.88 1.76
标本1-0(10) / P1 / P2
RMS Freq G1 Freq S Freq G2 G2/G1
32.26 66.34 20.83 9.65 1.78
MOLM-13 R30
标本1-0(3) / P1 / P2
RMS Freq G1 Freq S Freq G2 G2/G
33.52 71.13 17.20 9.78 1.76
标本1-0(7) / P1 / P2
RMS Freq G1 Freq S Freq G2 G2/G1
31.97 67.67 18.38 10.78 1.76
标本1-0(11) / P1 / P2
RMS Freq G1 Freq S Freq G2 G2/G1
38.48 72.60 17.64 7.62 1.79
MOLM-13 R50
标本1-0(4) / P1 / P2
RMS Freq G1 Freq S Freq G2 G2/
25.32 71.19 16.25 10.01 1.74
标本1-0(8) / P1 / P2
RMS Freq G1 Freq S Freq G2 G2/G
22.19 68.70 17.87 9.55 1.74
标本1-0(12) / P1 / P2
RMS Freq G1 Freq S Freq G2 G2/G1
29.41 68.51 20.64 8.29 1.78
MV4-11 S
标本1-0(1) / P1 / P2
RMS Freq G1 Freq S Freq G2 G2/G1
9.04 39.70 38.90 19.63 1.77
标本1-0(5) / P1 / P2
RMS Freq G1 Freq S Freq G2 G2/G1
14.03 43.11 36.29 18.72 1.76
标本1-0(9) / P1 / P2
RMS Freq G1 Freq S Freq G2 G2/G1
9.89 41.12 40.32 16.63 1.77
MV4-11R10
标本1-0(2) / P1 / P2
RMS Freq G1 Freq S Freq G2 G2/G1
5.04 48.17 37.64 9.97 1.84
标本1-0(6) / P1 / P2
RMS Freq G1 Freq S Freq G2 G2/G1
21.26 50.69 33.85 12.68 1.80
标本1-0(10) / P1 / P2
RMS Freq G1 Freq S Freq G2 G2/G1
17.59 55.11 24.84 16.37 1.78
MV4-11 R30
标本1-0(3) / P1 / P2
RMS Freq G1 Freq S Freq G2 G2/G1
5.61 56.54 25.22 13.88 1.79
标本1-0(7) / P1 / P2
RMS Freq G1 Freq S Freq G2 G2/G1
13.69 55.32 27.12 12.65 1.81
标本1-0(11) / P1 / P2
RMS Freq G1 Freq S Freq G2 G2/G1
14.63 48.83 35.51 12.62 1.80
MV4-11 R50
标本1-0(4) / P1 / P2
RMS Freq G1 Freq S Freq G2 G2/G1
9.70 53.73 22.93 15.28 1.79
标本1-0(8) / P1 / P2
RMS Freq G1 Freq S Freq G2 G2/G1
8.41 52.43 25.73 14.14 1.80
标本1-0(12) / P1 / P2
RMS Freq G1 Freq S Freq G2 G2/G1
7.77 49.89 22.03 15.71 1.76

Apoptosis

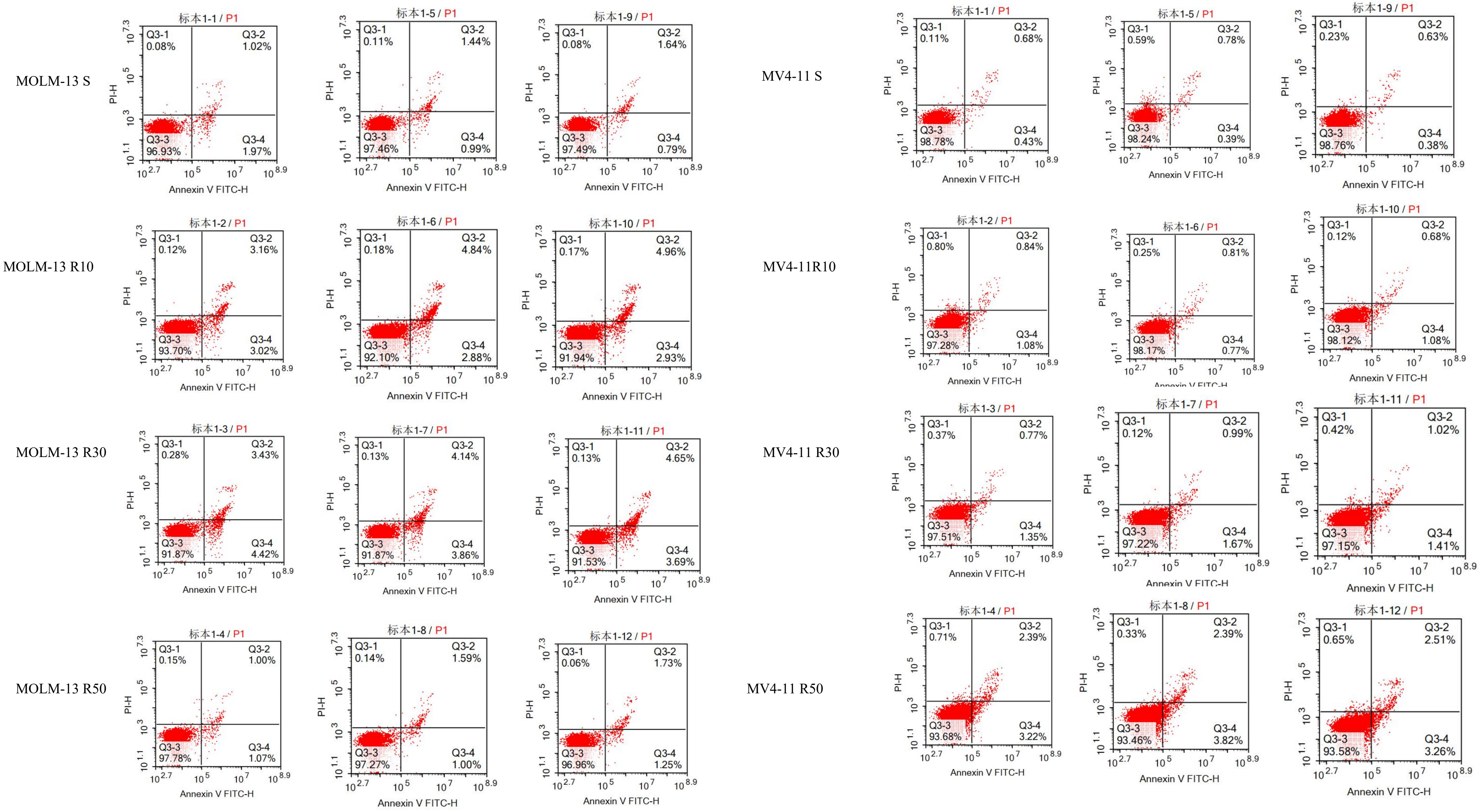
MOLM-13 S
标本1-1 / P1
Q3-1 0.08%
Q3-2 1.02%
Q3-3 96.93%
Q3-4 1.97%
标本1-5 / P1
Q3-1 0.11%
Q3-2 1.44%
Q3-3 97.46%
Q3-4 0.99%
标本1-9 / P1
Q3-1 0.08%
Q3-2 1.64%
Q3-3 97.49%
Q3-4 0.79%
MV4-11 S
标本1-1 / P1
Q3-1 0.11%
Q3-2 0.68%
Q3-3 98.78%
Q3-4 0.43%
标本1-5 / P1
Q3-1 0.59%
Q3-2 0.78%
Q3-3 98.24%
Q3-4 0.39%
标本1-9 / P1
Q3-1 0.23%
Q3-2 0.63%
Q3-3 98.76%
Q3-4 0.38%
MOLM-13 R10
标本1-2 / P1
Q3-1 0.12%
Q3-2 3.16%
Q3-3 93.70%
Q3-4 3.02%
标本1-6 / P1
Q3-1 0.18%
Q3-2 4.84%
Q3-3 92.10%
Q3-4 2.88%
标本1-10 / P1
Q3-1 0.17%
Q3-2 4.96%
Q3-3 91.94%
Q3-4 2.93%
MV4-11R10
标本1-2 / P1
Q3-1 0.80%
Q3-2 0.84%
Q3-3 97.28%
Q3-4 1.08%
标本1-6 / P1
Q3-1 0.25%
Q3-2 0.81%
Q3-3 98.17%
Q3-4 0.77%
标本1-10 / P1
Q3-1 0.12%
Q3-2 0.68%
Q3-3 98.12%
Q3-4 1.08%
MOLM-13 R30
标本1-3 / P1
Q3-1 0.28%
Q3-2 3.43%
Q3-3 91.87%
Q3-4 4.42%
标本1-7 / P1
Q3-1 0.13%
Q3-2 4.14%
Q3-3 91.87%
Q3-4 3.86%
标本1-11 / P1
Q3-1 0.13%
Q3-2 4.65%
Q3-3 91.53%
Q3-4 3.69%
MV4-11 R30
标本1-3 / P1
Q3-1 0.37%
Q3-2 0.77%
Q3-3 97.51%
Q3-4 1.35%
标本1-7 / P1
Q3-1 0.12%
Q3-2 0.99%
Q3-3 97.22%
Q3-4 1.67%
标本1-11 / P1
Q3-1 0.42%
Q3-2 1.02%
Q3-3 97.15%
Q3-4 1.41%
MOLM-13 R50
标本1-4 / P1
Q3-1 0.15%
Q3-2 1.00%
Q3-3 97.78%
Q3-4 1.07%
标本1-8 / P1
Q3-1 0.14%
Q3-2 1.59%
Q3-3 97.27%
Q3-4 1.00%
标本1-12 / P1
Q3-1 0.06%
Q3-2 1.73%
Q3-3 96.96%
Q3-4 1.25%
MV4-11 R50
标本1-4 / P1
Q3-1 0.71%
Q3-2 2.39%
Q3-3 93.68%
Q3-4 3.22%
标本1-8 / P1
Q3-1 0.33%
Q3-2 2.39%
Q3-3 93.46%
Q3-4 3.82%
标本1-12 / P1
Q3-1 0.65%
Q3-2 2.51%
Q3-3 93.58%
Q3-4 3.26%
PI-H
Annexin V FITC-H

WB: mice tumour

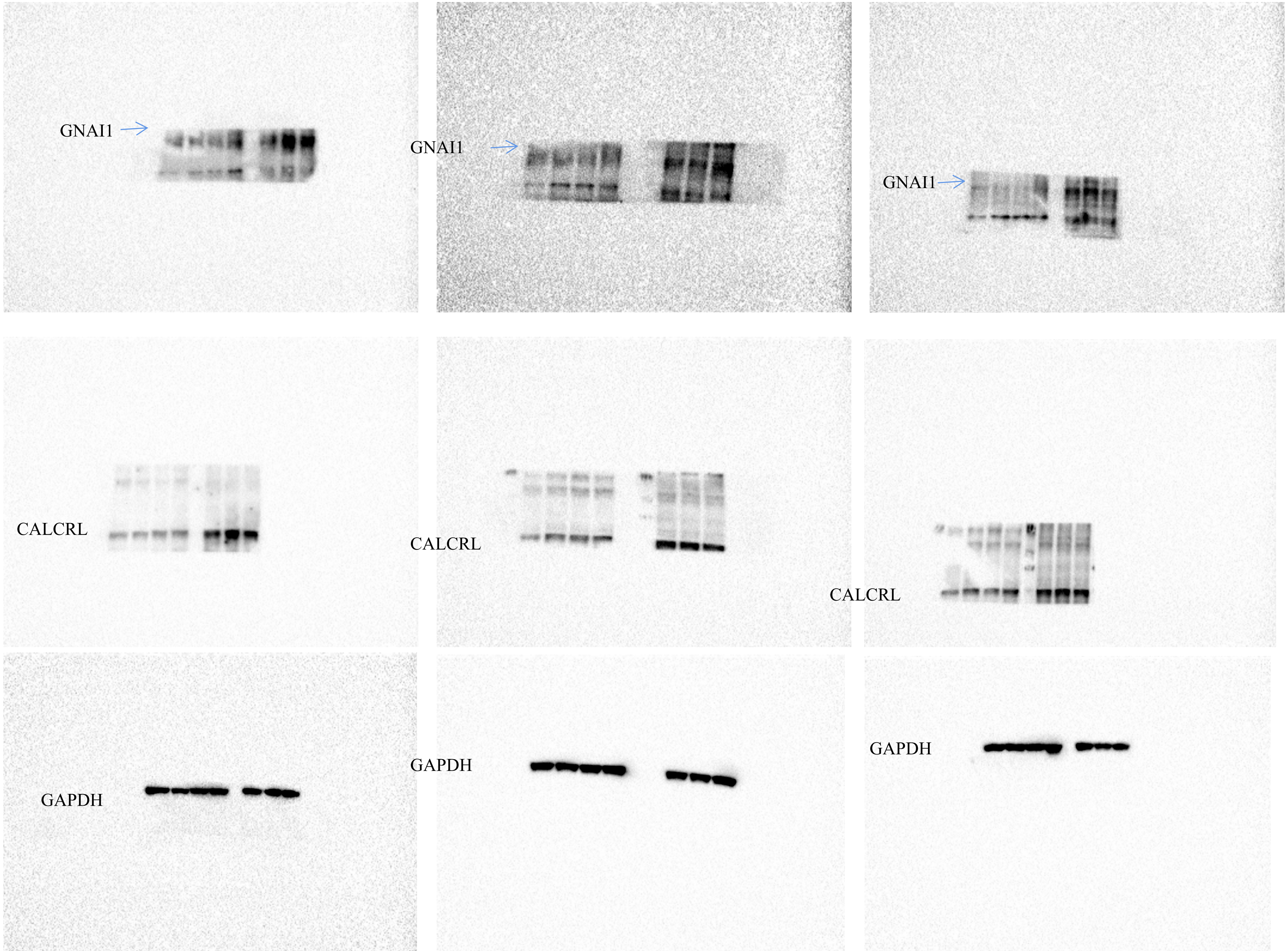

WB: cell lines

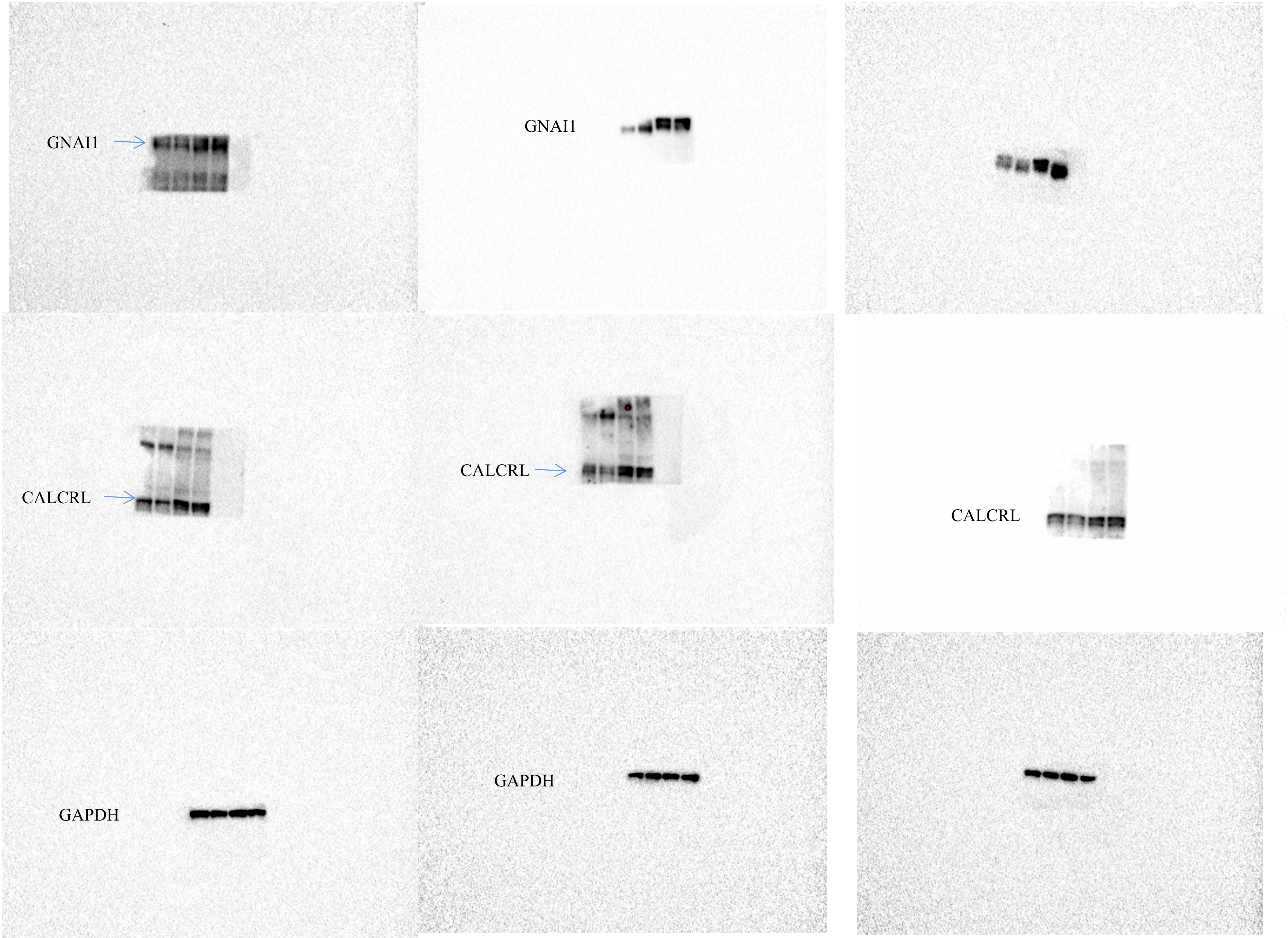

Mice tumour HE: MV4-11 S(Three mice)

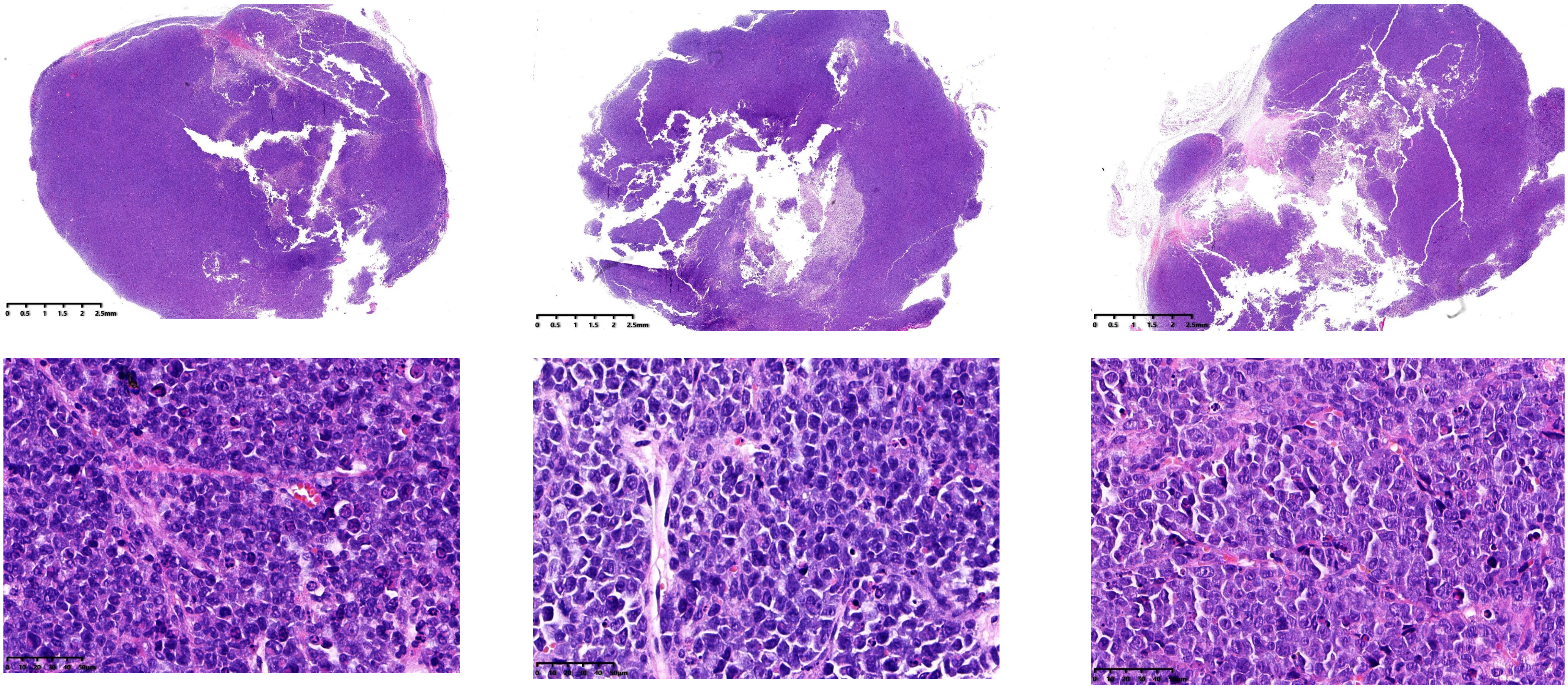

Mice tumour HE: MV4-11 R50(Three mice)

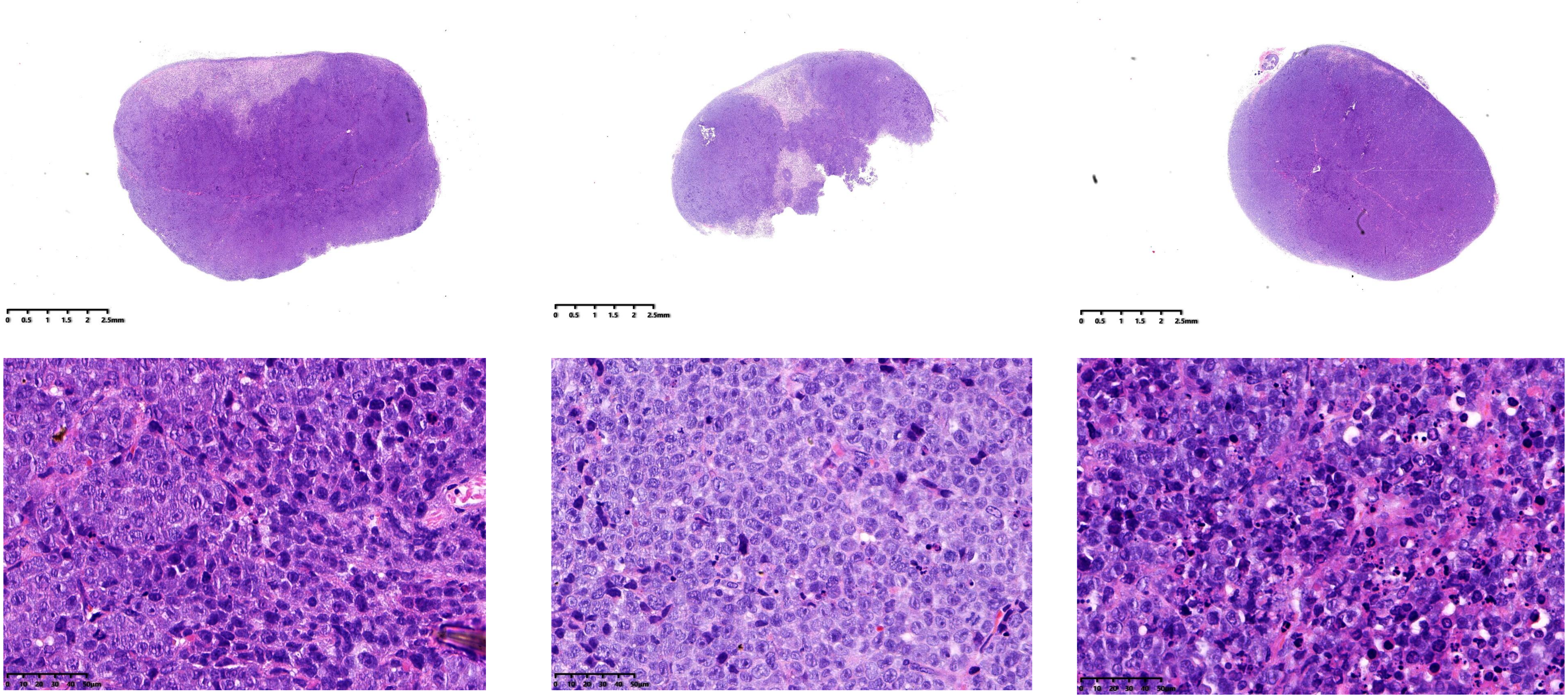

Part of the Cell Identification Certificate：

THP-1:

2. database annotation

Figure 1. STR matching analysis

EV	Cell No.	Cell name	Locus names								
			D5S818	D13S317	D7S820	D16S539	VWA	TH01	AM	TPOX	CSF1PO
	Query (Your Cell)		11,12	13,13	10,10	11,12	16,16	8,9.3	X,Y	8,11	11,13
1.0(36/36)	TIB-202	THP-1	['11', '12']	['13', '13']	['10', '10']	['11', '12']	['16', '16']	['8', '9.3']	['X', 'Y']	['8', '11']	['11', '13']

Note: The STR online match analysis of the test cell against DSMZ database, showing cell number (Cell No.) and cell name.

MV4-11:

2. database annotation

Figure 1. STR matching analysis

EV	Cell No.	Cell name	Locus names								
			D5S818	D13S317	D7S820	D16S539	VWA	TH01	AM	TPOX	CSF1PO
	Query (Your Cell)		11,12	13,13	8,9	11,12	14,15	8,9.3	X,Y	8,11	10,12
1.00(36/36)	102	MV4-11	11,12	13,13	8,9	11,12	14,15	8,9.3	X,Y	8,11	10,12
1.00(36/36)	CRL-9591	MV-4-11	11,12	13,13	8,9	11,12	14,15	8,9.3	X,Y	8,11	10,12

MOLM-13:

2. database annotation

Figure 1. STR matching analysis

EV	Cell No.	Cell name	Locus names								
			D5S818	D13S317	D7S820	D16S539	VWA	TH01	AM	TPOX	CSF1PO
	Query (Your Cell)		10,11	10,11	10,12	10,11	16,17	7,7	X,Y	8,8	10,12
1.0(36/36)	554	MOLM-13	['10', '11']	['10', '11']	['10', '12']	['10', '11']	['16', '17']	['7', '7']	['X', 'Y']	['8', '8']	['10', '12']

Note: The STR online match analysis of the test cell against DSMZ database, showing cell number (Cell No.) and cell name.

KG-1:

2. database annotation

Figure 1. STR matching analysis

EV	Cell No.	Cell name	Locus names								
			D5S818	D13S317	D7S820	D16S539	VWA	TH01	AM	TPOX	CSF1PO
	Query (Your Cell)		13,13	11,12	8,10	10,11	14,19	7,8	X,Y	7,9	7,7
1.00(36/36)	14	KG-1	13,13	11,12	8,10	10,11	14,19	7,8	X,Y	7,9	7,7

Note: The STR online match analysis of the test cell against DSMZ database, showing cell number (Cell No.) and cell name.

KASUMI-1:

2. database annotation

Figure 1. STR matching analysis

EV	Cell No.	Cell name	Locus names								
			D5S818	D13S317	D7S820	D16S539	VWA	TH01	AM	TPOX	CSF1PO
	Query (Your Cell)		9,11	11,13	8,11	9,12	14,14	6,9	X,X	8,9	10,12
1.00(36/36)	220	KASUMI-1	9,11	11,13	8,11	9,12	14,14	6,9	X,X	8,9	10,12
1.00(36/36)	CRL-2724	Kasumi-1	9,11	11,13	8,11	9,12	14,14	6,9	X,X	8,9	10,12

Note: The STR online match analysis of the test cell against DSMZ database, showing cell number (Cell No.) and cell name.

HL-60:

2. database annotation

Figure 1. STR matching analysis

EV	Cell No.	Cell name	Locus names								
			D5S818	D13S317	D7S820	D16S539	VWA	TH01	AM	TPOX	CSF1PO
	Query (Your Cell)		12,12	8,11	11,12	11,11	16,16	7,8	X,X	8,11	13,14
1.0(36/36)	3	HL-60	['12', '12']	['8', '11']	['11', '12']	['11', '11']	['16', '16']	['7', '8']	['X', 'X']	['8', '11']	['13', '14']

Note: The STR online match analysis of the test cell against DSMZ database, showing cell number (Cell No.) and cell name.
